# Supplementary material for: Bumblebees minimize control challenges by combining active and passive modes in unsteady winds
Source: Sci Rep. 2016 Oct 18;6:35043. doi: 10.1038/srep35043 (PMC5067513; doi:10.1038/srep35043)
Supplement: Supplementary Information [file srep35043-s1.pdf]

Bumblebees minimize control challenges by combining active and passive modes in unsteady winds.

Supplementary material

Sridhar Ravi*[1,2], Dmitry Kolomenskiy[1], Thomas Engels[3], Kai Schneider[4], Chun Wang[2], Jörn Sesterhenn[3] and Hao Liu[1,5]

[1]Graduate School of Engineering, Chiba University, Chiba, Japan
[2]School of Aerospace Mechanical and Manufacturing Engineering, RMIT University, Melbourne, Australia
[3]ISTA, Technische Universität Berlin, Berlin, Germany
[4]I2M - UMR 7373 - CNRS, Centre de Mathématiques et d'Informatique, Aix-Marseille Université, Marseille, France
[5]Shanghai-Jiao Tong University and Chiba University International Cooperative Research Centre (SJTU-CU ICRC), Shanghai, China

September 21, 2016

S1 Numerical simulation

The the flow configuration used for the numerical simulation of uncontrolled flight is visualized in figure SF1, and a sample flow visualization is shown in figure 3. This section provides an extended discussion of the results of the numerical simulation.

Figure SF2 displays the time evolution of the accelerations and the displacements of the body from the beginning of the simulation. The insect in initially at rest, in-line with the cylinder, and its roll angle is zero. The asymmetry in the wake of the cylinder

*Corresponding Author: sridhar.ravi@rmit.edu.au

increases in time. The vortex street begins to have a significant influence on the insect after $t = 50$ ms.

The profiles of linear and angular accelerations are shown in figure SF2(*top row*) with blue dashed lines. They both consist of slow quasi-periodic oscillations superimposed with a high-frequency noisy component. The frequency of quasi-periodic oscillations is about 24 Hz, which is the vortex shedding frequency of the cylinder. This acceleration is a direct consequence of the periodic sidewash by the vortex street. The high-frequency oscillations occur on the time scale even shorter than the wingbeat frequency. They are due to the aerodynamic interaction between the wings and the small-scale turbulence in the wake. In the wind tunnel experiment, the time resolution of the inverse dynamics analysis is limited by the frame rate of the video sequences. Therefore, in order to establish a comparison between the numerical simulation and the experiment, we applied the 4th order Butterworth low-pass filter at 30 Hz to the time series of the accelerations. The results are shown with the light blue lines. We focus on the time interval of three shedding periods (125 ms) after the beginning of the quasi-periodic state at $t = 95$ ms.

The time evolution of the lateral displacement and of the roll angle is shown in figure SF2(*bottom row*). Note that oscillations are only visible at the von Kármán shedding frequency and not at the flapping frequency, because time integration has a low-pass filtering effect. It is also important to keep in mind that we are considering a fluid-structure interaction problem which does not necessarily have a time-periodic so-

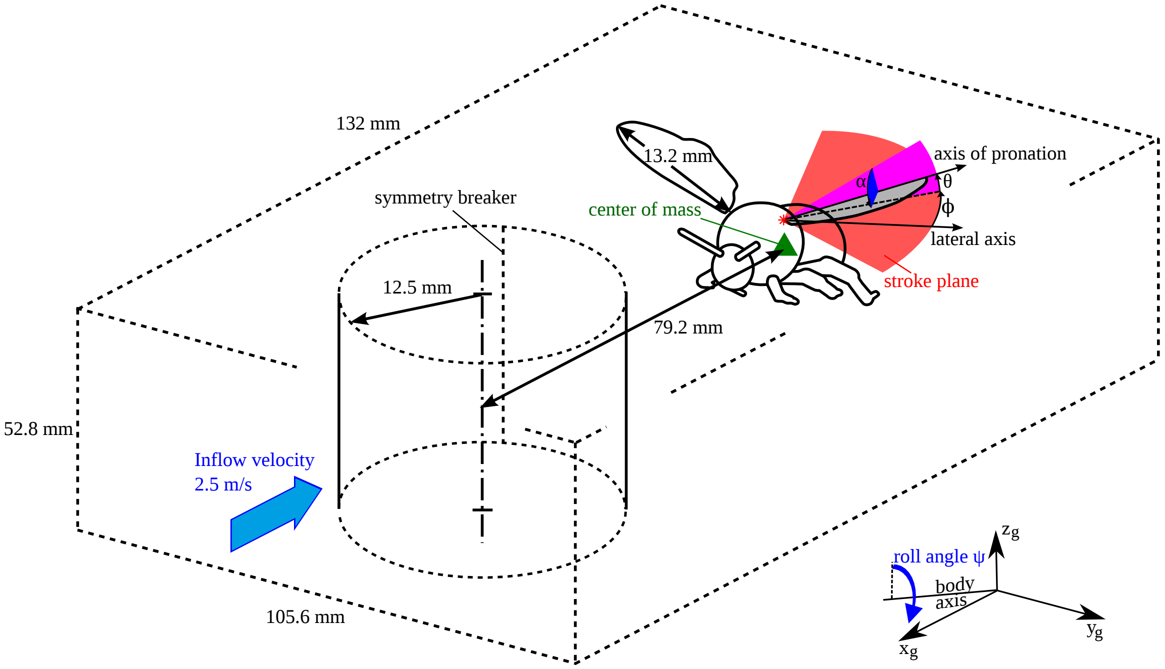

Figure SF1: Schematic drawing of the bumblebee model. See Materials and Methods section for the description of the numerical setup.

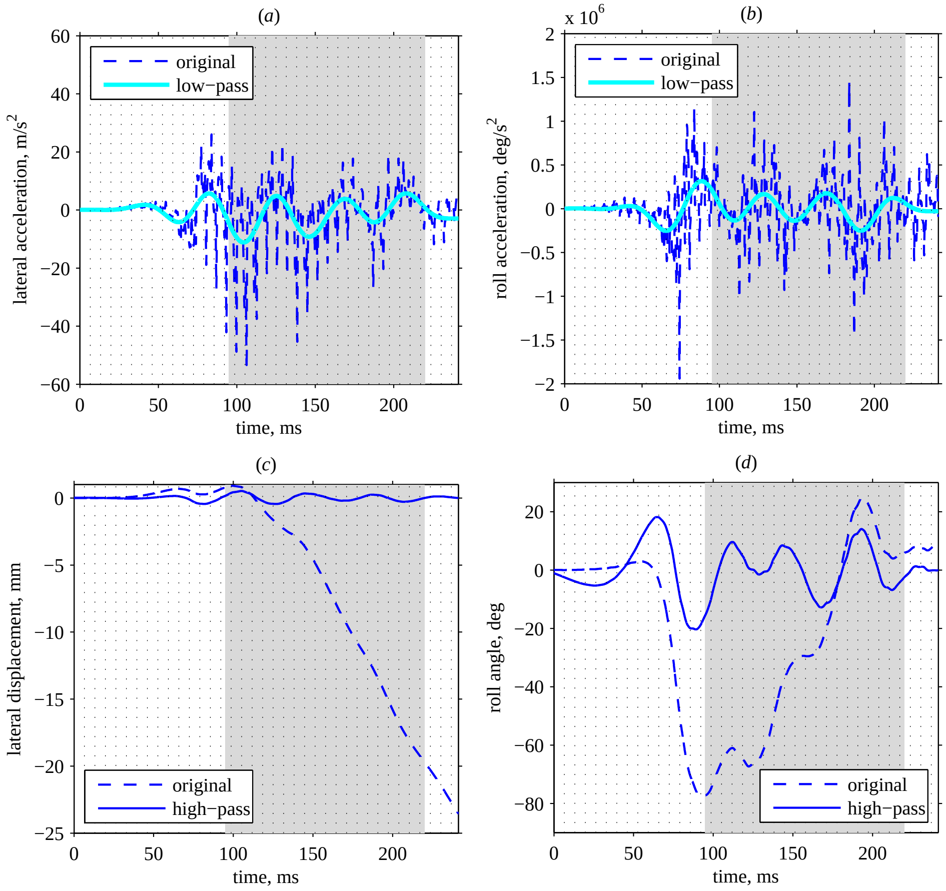

Figure SF2: Time evolution of the accelerations and displacements in the numerical simulation. Top row: lateral acceleration (a) and roll acceleration (b). Bottom row: lateral displacement (c) and roll angle (d). The gray shaded area indicates the time window used for comparison with the experiment. Vertical dotted lines indicate the wingbeat periods.

lution. An impulsive start from the quescent flow initial condition and turbulence in the wake of the cylinder produce a slight imbalance between the consequent cycles of right and left sidewash during the beginning of the motion. These effects result in a slow large-amplitude drift of the insect model. However we are only interested in a time interval shorter than the slow casting motion, because the bees can definitely control their king kinematics at that long time scale. Therefore we apply the 4th order Butterworth high-pass filter at 10 Hz to the time series of lateral and angular displacement in order to focus on the quasi-periodic oscillations at the von Kármán shedding frequency. The result is shown with solid lines in figure SF2(*bottom row*). Again, we focus on a time subsequence of 125 ms to compare with the experiment.

S2 Reduced-order modeling of roll-sideslip oscillations

In this section we derive the equations that describe the dynamics of the low-frequency motion (casting) and high-frequency motion. The time series of accelerations and of the roll angle, obtained in the experiments, show scale separation between slow casting motion and rapid buffeting at the von Kármán shedding frequency. We hypothesize that these are two conceptually different modes:

- *Active (helicopter) mode*, which is a voluntary low-frequency casting motion;
- *Passive mode*, which is an unvoluntary passive high-frequency advection motion.

We propose idealized analytical models of these two modes.

S2.1 Active (helicopter) mode

The insect drives this motion by asymmetrically changing the kinematics of its right and left wings. Thus, the insect controls the roll moment M_x. Let us assume it varies in time as a sine wave with given frequency f_c,

$$M_x(t) = \hat{M} \sin(2\pi f_c t). \tag{1}$$

The body coordinate system is shown in figure SF3. To obtain the time evolution of the roll angle $\psi(t)$, we integrate the equation of roll motion,

$$I_{xx}\ddot{\psi} = M_x(t), \tag{2}$$

where I_{xx} is the moment of inertia. Using the time periodicity condition $\psi(t+1/f_c) = \psi(t)$, we obtain

$$\psi = \hat{\psi} \sin(2\pi f_c t), \quad \text{where} \quad \hat{\psi} = -\frac{\hat{M}}{4\pi^2 f_c^2 I_{xx}}. \tag{3}$$

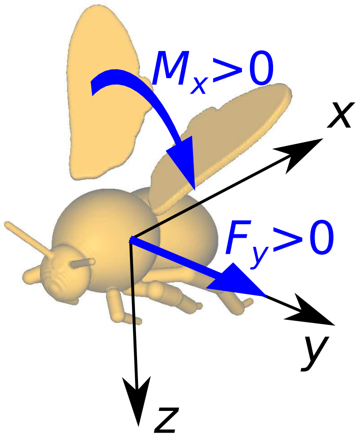

Figure SF3: Coordinate system attached to the insect body, used in the reduced-order models. Blue arrows show the positive directions of roll torque and lateral force.

When the body roll angle ψ is non-zero, the lateral force F_y becomes non-zero. Let us assume that ψ varies in a range where we can use the small angle approximation (see figures 1 and 2 of the article). Since the vertical aerodynamic force supports the body weight, $F_z = mg$, we obtain

$$F_y = F_z \tan\psi \sim mg\psi. \tag{4}$$

Note that the aerodynamic force due the asymmetric wing kinematics of the right and left wings may also contribute to the lateral force. In our simple model we neglect this secondary effect. Using (3), we obtain

$$F_y = \hat{F}\sin(2\pi f_c t), \quad \text{where} \quad \hat{F} \sim -\frac{mg\hat{M}}{4\pi^2 f_c^2 I_{xx}}. \tag{5}$$

Finally, we integrate the equation of motion,

$$m\ddot{y} = F_y(t), \tag{6}$$

to obtain the time evolution of the lateral displacement,

$$y = \hat{y}\sin(2\pi f_c t), \quad \text{where} \quad \hat{y} \sim \frac{g\hat{M}}{16\pi^4 f_c^4 I_{xx}}. \tag{7}$$

The motion described by (7) and (3) is visualized in figure 6(a). Note that while $\hat{y}$ is positive, $\hat{\psi}$ is negative. This implies that the leftmost lateral position (maximum positive y) corresponds to the maximum roll to the right (maximum negative ψ). From (7) and (3) we also obtain

$$\frac{\hat{y}}{\hat{\psi}} = -\frac{g}{4\pi^2 f_c^2}. \tag{8}$$

For example, at $f_c = 2$ Hz, we obtain $\hat{y}/\hat{\psi} = -62$ mm/rad. Hence, the roll amplitude of $\hat{\psi} = 20°$ would be accompanied by $\hat{y} = -22$ mm lateral displacement amplitude, see Supplementary Video 3 for visualization. Whereas low-frequency casting motion only requires relatively small roll, high-frequency casting motion with the same lateral displacement amplitude would require unrealistically large roll angle.

Finally, for comparison with the experiment, it is important to notice that the gain coefficient in this model is a constant,

$$G = \frac{F_y}{m\psi} = g \approx 9.81 \text{ m/s}^2/\text{rad}. \tag{9}$$

S2.2 Passive mode

In this case, we suppose that the insect is only driven by the aerodynamic drag due to the external flow. A major simplifying assumption is to neglect the time variation of the shape of the insect due to wing flapping. This approximation is common in the studies on the dynamics of flapping flight (*cf.* [3]), motivated by the "averaged" models considered in classical helicopter theories (see, *e.g.*, [1, 2]). Since the flapping frequency is large compared to the frequency of the two modes that we are concerned with, only the wingbeat-averaged forces have significant effect on the body dynamics. In addition, we neglect any possible nonlinear coupling between the lower-frequency and the high-frequency motions. Comparison between the numerical simulation and the experiments suggests that, essentially, the high-frequency mode is insensitive to the low-frequency dynamics, as long as the bee stays in the vortex street.

The insect model is placed in a von Kármán street, which induces periodic side wind with frequency f_v. Let us assume that, in the oscillating side wind, the elementary aerodynamic force acting on a surface element $\boldsymbol{\sigma}$ at a point $\boldsymbol{\xi}$ is equal to

$$\boldsymbol{q}(\boldsymbol{\xi}, t) = \hat{\boldsymbol{q}}(\boldsymbol{\xi}) \sin(2\pi f_v t). \tag{10}$$

By integration over the surface of the body, we obtain the force

$$\boldsymbol{F}(t) = \int_\Sigma \hat{\boldsymbol{q}}(\boldsymbol{\xi}) \sin(2\pi f_v t) \mathrm{d}\sigma(\boldsymbol{\xi}) \tag{11}$$

and the torque

$$\boldsymbol{M}(t) = \int_\Sigma ((\boldsymbol{\xi} - \boldsymbol{x}_{cg}) \times \hat{\boldsymbol{q}}(\boldsymbol{\xi})) \sin(2\pi f_v t) \mathrm{d}\sigma(\boldsymbol{\xi}), \tag{12}$$

where Σ is the surface of the insect and $\boldsymbol{x}_{cg}$ is its center of gravity. We are only interested in the y component of $\boldsymbol{F}(t)$ and x component of $\boldsymbol{M}(t)$. Let us define the moment arm L,

$$L = M_x(t)/F_y(t). \tag{13}$$

The torque is positive if the force is positive and the center of pressure is situated above the center of gravity. We approximate L by a constant $\overline{L}$. A practical estimate of $\overline{L}$ is

given later in the text. We obtain the following approximate formulae for the lateral force and roll torque:

$$F_y = \hat{F}\sin(2\pi f_v t), \quad \text{where} \quad \hat{F} = \int_\Sigma \hat{q}_y(\boldsymbol{\xi})\mathrm{d}\sigma(\boldsymbol{\xi}) \tag{14}$$

and

$$M_x = \hat{M}\sin(2\pi f_v t), \quad \text{where} \quad \hat{M} = \hat{F}\overline{L}. \tag{15}$$

From the solid body dynamics equations (2) and (6), requiring that the motion is periodic in time, we find

$$y = \hat{y}\sin(2\pi f_v t), \quad \text{where} \quad \hat{y} = -\frac{\hat{F}}{4\pi^2 f_v^2 m} \tag{16}$$

and

$$\psi = \hat{\psi}\sin(2\pi f_v t), \quad \text{where} \quad \hat{\psi} = -\frac{\hat{F}\overline{L}}{4\pi^2 f_v^2 I_{xx}}. \tag{17}$$

This motion is visualized in figure 5(*b*). Note that $\hat{y}$ and $\hat{\psi}$ have the same sign now. This implies that the leftmost lateral position (maximum positive y) corresponds to the maximum roll to the left (maximum positive ψ).

It is instructive to write the proportionality between the lateral displacement and the roll angle,

$$\frac{\hat{y}}{\hat{\psi}} = \frac{I_{xx}}{\overline{L}m}. \tag{18}$$

It does not depent of the frequency, in contrast to (8), but it depends on the shape of the body. In the experiments, the average body mass was equal to $m = 165$ mg, moment of inertia $I_{xx} = 8.49 \cdot 10^{-10}$ kgm^2, and the vortex shedding frequency $f_v = 23$ Hz. The moment arm is estimated to be equal to $\overline{L} = 1.4$ mm, using the numerical simulation. We thus obtain $\hat{y}/\hat{\psi} = 3.7$ mm/rad. The roll angle amplitude of 20° corresponds now to the lateral displacement amplitude of only 1.3 mm, which is much less than during casting discussed in the previous section. For the roll angle amplitude of 7°, as in the experiments, the lateral displacement amplitude is less than 0.5 mm, see Supplementary Video 4 for visualization.

Finally, let us calculate the proportionality coefficient

$$\Pi = \frac{F_y}{m\psi} = -\frac{4\pi^2 f_v^2 I_{xx}}{\overline{L}m} \approx -76.6 \text{ m/s}^2\text{/rad}, \tag{19}$$

which is a counterpart of the gain G defined by (9).

S3 Additional numerical simulations at lower Reynolds number

We have conducted additional numerical simulations to test sensitivity of our results to different parameters. The new computations include two different configurations. The first is a series of runs with the initial lateral position of the c.g., y_0, varied from -2.64 mm to +2.64 mm with respect to the original position $y_0 = 0$. The viscosity of the air was increased by a factor of 4 compared to the value used in the original simulation. *I.e.*, we reduced the Reynolds number by a factor of 4. This allowed substantially reduce the computational cost by reducing the number of grid points to $960 \times 768 \times 384$ and increasing the volume penalization parameter to 10^{-3}. It is known that the aerodynamic force coefficients depend on the Reynolds number as $\mathcal{O}(Re^0)$ in our range of Re, therefore, these lower-Reynolds number computations can provide qualitative estimates and trends.

The results of five new simulations are shown in figure SF4 in the end of this document. The original simulation is also shown on the same figure, for reference. The low-frequency motion (< 10 Hz) turns out to be sensitive to Re and to y_0, but in all cases the lateral displacement (panel (f)) increases over time, *i.e.*, the insect drifts sideways. The high-frequency motion (> 10 Hz) is remarkably similar in all cases, which confirms our theoretical model of the passive mode.

The time interval of 0.2 s may be too short to conclude about the low-frequency dynamics. For this purpose, figure SF5 shows another low-Re simulation with larger cylinder (43 mm) and longer time evolution. The body is fully fixed during the first half of the simulation, then the two degrees of freedom are released. This helps to avoid the initial "kick-start" from the first vortex in the vortex street, which is stronger than the subsequent vortices. Now the time interval is sufficiently long to analyze the low-frequency motion (panels (c) and (f)). We clearly see diverging oscillatory behavior. The roll angle eventually increases to unrealistically large values. We conclude that, in all cases, the high frequency motions are relatively small in magnitude and almost periodic, but lower-frequency instabilities grow over time.

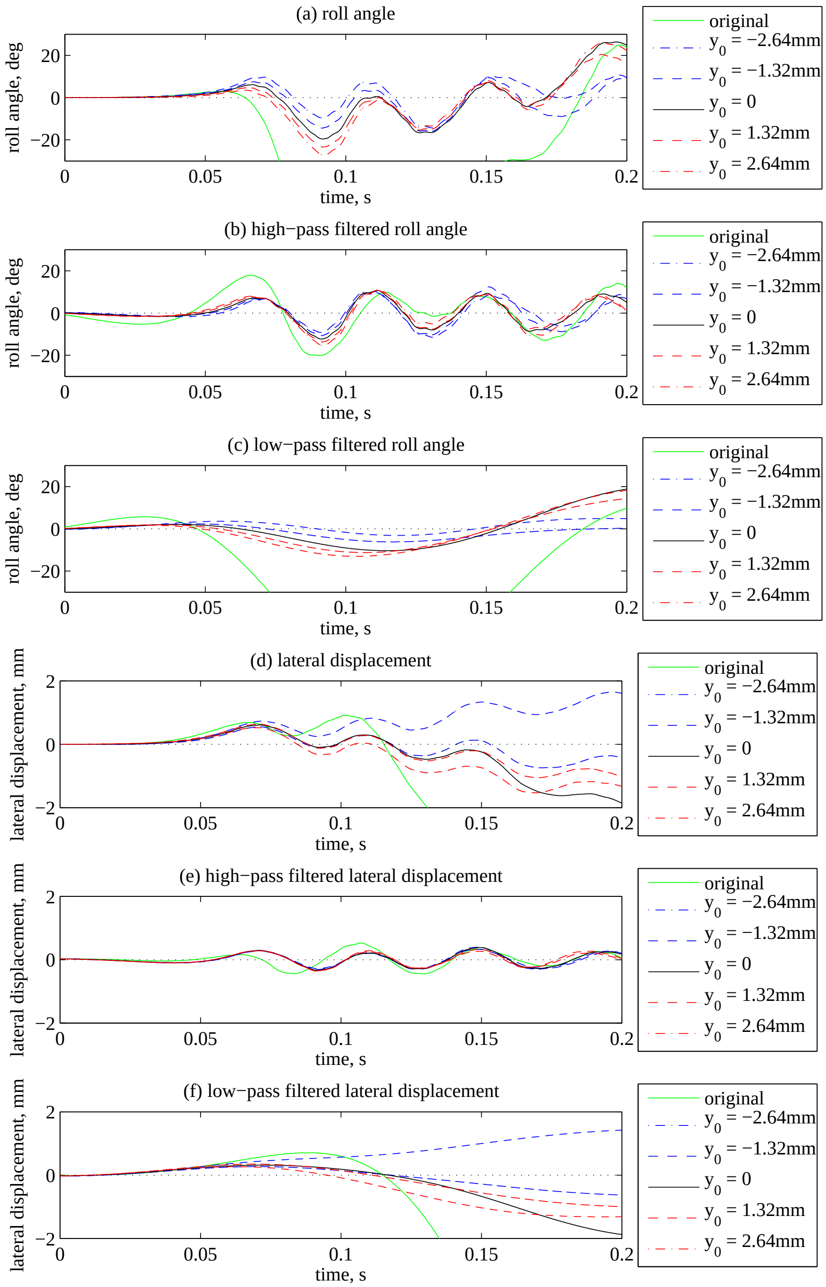

Figure SF4: Effect of varying the initial lateral position of the insect. Time evolution of the roll angle (a), high-pass filtered roll angle (b), low-pass filtered roll angle (c), lateral displacement (d), high-pass filtered lateral displacement (e), low-pass filtered lateral displacement (f).

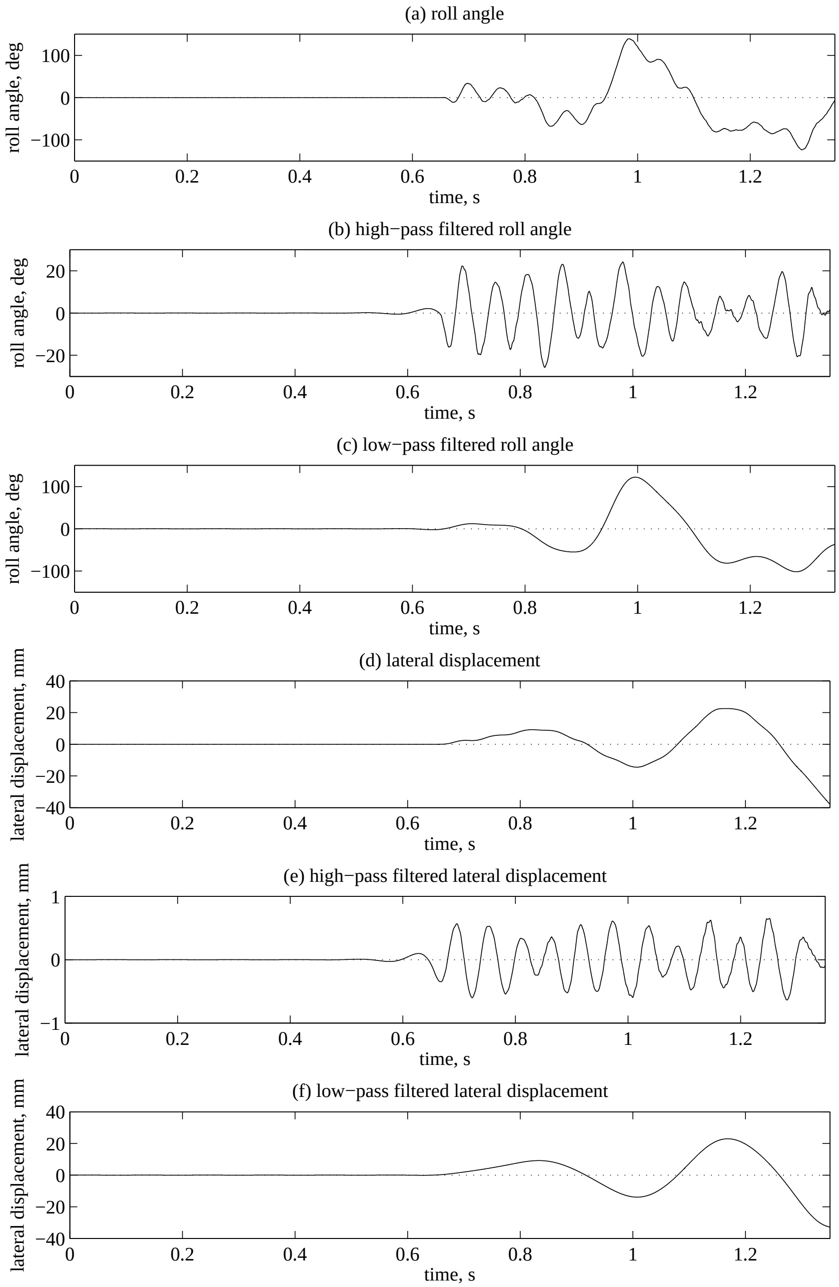

Figure SF5: Long flight in the wake of a large cylinder. Time evolution of the roll angle (a), high-pass filtered roll angle (b), low-pass filtered roll angle (c), lateral displacement (d), high-pass filtered lateral displacement (e), low-pass filtered lateral displacement (f).

S4 Supplementary Videos

- **Supplementary Video 1**: Sample video of a bumblebee flying in the wake of a cylinder. Colors highlight the flight trajectory of the bee.
- **Supplementary Video 2**: View of the numerical simulation of a bumblebee with no active flight control flying in the wake of a cylinder. Isosurfaces of dimensionless vorticity magnitude are shown as, light blue $|\omega| = 5$, red $|\omega| = 40$.
- **Supplementary Video 3**: Animation of the body motion produced by a bumblebee while performing active casting maneuvers. Red dot shows the position of the center of gravity, green lines visualize the laboratory frame of reference.
- **Supplementary Video 4**: Animation of the body motion experienced by a bumblebee while flying in unsteady winds - buffeting motion. Red dot shows the position of the center of gravity, green lines visualize the laboratory frame of reference.

References

[1] K. Hohenemser. Über die dynamische Stabilität des Hubschraubers mit angelenkten Flügeln. *Ingenieur-Archiv*, 9(6):419–428, 1938.

[2] K. Hohenemser. Dynamic stability of a helicopter with hinged rotor blades. *NACA Technical Memorandum*, 907:1–19, 1939.

[3] G. K. Taylor and A. L. R. Thomas. Animal flight dynamics II. Longitudinal stability in flapping flight. *Journal of Theoretical Biology*, 214(3):351–370, 2002.
